# Medical Cases, with Occasional Remarks

**Published:** 1819-04-01

**Authors:** 


### *MEDICAL CASES, with occasional REMARKS.*

By Dr. Carbutt.

( Continued from Page 168. )

Case 2. *Typhus Fever, combined with Pneumonia; and successfully treated, with copious Blood-letting.*

THE following Case is given nearly *verbatim* from my notes, taken at the time; a method of publication which I much prefer to that of dressing up Cases for the purpose.

August 12th, 1818. John Watkin, a boatman, aged twenty-three years, was visited by me, at his residence, on behalf of the Manchester Infirmary. I found that he had been ill six weeks, but had left off work only fourteen days. He had been engaged in cleaning a canal, where he had, of course, been much exposed to damp. Since his illness supervened, he has complained chiefly of pain in his back, sides and head; the pain in his head he described as being much increased by bending forward, and still more by stooping. Since he left his work, has been altogether powerless in his limbs; has had considerable cough, and some expectoration. Has been attended by a respectable surgeon, who ordered nothing but the pediluvium every night, and a medicine, the phial of which smells strongly of ether. I ordered him to be conveyed to the House of Recovery.

The symptoms at present are as follow:—A remarkable degree of deafness, which has come on since he left work; face flushed, its expression anxious; the conjunctiva suffused with blood; tongue brown and parched; much thirst; respiration hurried, and he complains of pain in the thorax; skin dry and hot; pulse hardly perceptible, about 108 or 110, as nearly as could be counted.

I ordered a vein to be opened, and stood by to observe the effects. He bore the loss of sixteen ounces of blood, which was the exact quantity drawn, without any faintness, and without any change in the pulse. He seemed, however, in some measure, relieved.

℞. Hydrargyri submuriatis gr. x; jalapæ radicis contritæ ℈j, Misce. Sit dosis statim sumenda.

℞. Infusi sennæ f ℥iiß; tincturæ sennæ f ℥ß; potassæ tartratis ℥j; aquæ menthæ viridis f ℥ij; aquæ q. s. e. ut fiant f ℥viij. Sumatur cyathus singulis semihoris donec alvus soluta sit. Bibat decoctum hordei ad libitum.

13th. Manè. Coagulum of the blood neither buffy nor cupped; serum in larger proportion than usual, turbid, and of a greenish hue. Copious alvine evacuations, chiefly liquid, and of a dark, unhealthy appearance. Considerable expectoration, and the mucus rather puriform. The deafness, we think, somewhat diminished; respiration improved. The other symptoms nearly as yesterday.

Applicentur pectori cucurbitulæ cum ferro, ut extrahantur sanguinis f ℥xij. Postea imponatur emplastrum lyttæ amplum.

℞. Hydrargyri submuriatis gr. xij; camphoræ, ope spiritûs rectificati in pulverem tritæ, gr. vj; confectionis rosæ caninæ, q. s. s. contunde et divide in pilulas sex; sumatur una quartâ quâque horâ.

℞. Potassæ subcarbonatis ʒiij; acidi citrici ʒiiß; aquæ f ℥xij, Misce. Sumat. æger f ℥iß. quartis horis.

13th. Vesperi. Respiration more embarrassed.

Mittantur sanguinis f ℥x. Continuentur pilulæ hydrargyri submuriatis cum camphorâ, et mistura potassæ citratis.

℞. Confectionis sennæ ℥iß; oxymellis scillæ f ʒiij; mucilaginis acaciæ, syrupi papaveris, singulorum f ʒvj; misce. Sumatur f ʒj. subinde, urgente tussi.

14th. Manè. Bowels open; evacuations as before; urine limpid, and of the same hue as in health. Cough and expectoration continue; skin rather moist; pulse 112, excessively weak; thirst continues; tongue parched and brown. Deafness continues.

Mittantur sanguinis f ℥xij.*

---

* I may mention once for all, that the quantity of blood to be taken was not exactly prescribed in any instance in this case, but left to be regulated by the effect produced; the blank in my journal was then filled up by the gentleman who drew the blood.

Eat in balneum calidum ad grad. 100^mum semel. Sumat pilulam hydrargyri submuriatis cum camphorâ unam tertiis horis, et misturam potassæ citratis sextis horis; et confectionem compositam ut antea.

14th. Vesperi. After the bleeding and the warm bath, he slept for about three hours, and perspired profusely. Cough less troublesome, expectoration freer, and repiration somewhat improved. Tongue moist, countenance less flushed, and the expression less anxious. Has had five liquid stools in the course of the day. It is now discovered that there is, in both arms, an extraordinary distribution of the radial artery, which turns to the back of the wrist, much higher than usual. The pulsation which we felt was therefore, most probably, that of the volar branch of the radial artery, which, of course, is longer than usual in this instance. The pulsation of this branch was, as before mentioned, hardly perceptible. The pulse, now felt at the radial artery, where it crosses the radius, is 100, rather small and soft.

Continuentur pilulæ, mistura, confectio, et decoctum hordei.

15th. Manè. Tongue dry and brown in the middle, but moist at the edges; cough and expectoration freer; deafness continues; pulse 100; skin moist; bowels loose.

Continuentur omnia ut heri.

15th. Vesperi. Respiration is more frequent and anxious; pulse 112, and fuller; tongue very dry; some tenderness upon pressure of the abdomen; bowels exceedingly open; stools liquid.

Mittantur sanguinis f ℥xx. Repetatur balneum calidum hâc nocte. Omittantur pilulæ et mistura.

℞. Pilularum hydrargyri gr. xii; pulveris antimonialis gr. iv; confectionis rosæ caninæ q. s. s. contunde, et divide in pilulas iv. Sumatur una sextis horis.

℞. Liquoris antimonii tartarizati, f ℥j; tincturæ opii ♏. xxx; spiritûs lavandulæ compositi f ʒij; syrupi simplicis f ℥ß; aquæ menthæ viridis f ℥xiv. Sumatur f ℥iß sextis horis, sed alternis vicibus, intervallo horarum trium, quoad pilulas. Continuentur confectio et decoctum.

16th. Tongue rather moister; pulse 120, somewhat smaller; respiration easier. Other symptoms as before.

Continuentur pilulæ hydrargyri, mistura antimonialis, confectio, et decoctum.

17th. Tongue moist at the edges, with brown fur in the middle; respiration much easier; cough and expectoration abated; skin very moist; pulse 100, becoming rather full; bowels quite open; deafness continues. Has slept a good deal.

Continuentur omnia ut heri.

17th. Vesperi. Complains of pain in his breast. Other symptoms as before.

Mittantur sanguinis f ℥xiv. Continuentur pilulæ, mistura, confectio, et decoctum.

18th. Slightly delirious, which I think has been the case before, but not much observed. Pulse 100, rather full. Other symptoms unaltered.

Mittantur sanguinis f ℥xx.* Cont. omnia ut antea.

19th. Excessive diaphoresis. Other symptoms as before.

Diminuatur dosis misturæ antimonialis ad f ℥j. sextis horis. Pergant cætera ut antea.

20th. Pulse much smaller, 104; diaphoresis diminished; respiration easy; cough and expectoration diminished; sleeps much; urine more copious than before; and now, for the first time, high coloured and turbid. Last night and this morning slightly delirious.

Continuentur omnia.

21st. Deafness much diminished; diaphoresis continues; urine turbid. Appetite is beginning to return. Has no pain whatever: when asked how he felt himself, he answered, with a pleasant expression of countenance, that he was "mending apace."

Omittantur pilulæ et mistura.

℞. Massæ pilularum hydrargyri gr. xx; fingantur pilulæ octo. Sumantur duæ singulis noctibus.

℞. Senegæ radicis contusæ ℥j; glycyrrhizæ radicis contusæ ℥ß; aquæ Oij; decoque ad Oj; addens, sub finem coctionis, glycyrrhizæ radicem; tum cola. Sumantur f ℥ij. sextis horis.

22d. Pulse 100, small and soft; bowels open; fæces dark coloured and fœtid. Says that he feels very easy.—Pergat.

---

* At none of the times of drawing blood was it, after standing the usual period, either buffy or cupped.

23d. Diaphoresis abated; tongue dry; has considerable thirst; appetite returned; bowels open; fæces foul; urine high coloured, but not turbid. Hearing, in a great measure, returned; sleeps well, and has no pain.

Continuentur pilulæ, decoctum senegæ, et decoctum hordei.

25th. Pulse 100, rather feeble; tongue quite moist, slightly foul in the middle; skin moist; cough and expectoration moderate; urine and fæces as before.

Continuentur pilulæ hydragyri et decoctum senegæ.

26th.—29th. Continues to improve. Pergat.

30th. Pulse 84; expectoration much diminished. Pergat.

31st. His hearing is now perfectly restored. Tongue quite clean, but parched in the middle; bowels regular; fæces of a healthy colour and appearance; urine copious and high coloured, but not turbid; pulse 88, rather feeble; cough and expectoration much diminished; breathing quite easy; has no pain. Is very desirous of rising from his bed, and declares he is quite stout.

Continuentur pilulæ et decoctum.

September 2d. Pulse 80; urine quite natural.

Omittantur pilulæ. Continuentur decoctum.

4th. Pulse 72; cough and expectoration almost quite gone.

Continuentur decoctum. To sit up for an hour to-day.

5th. Pulse 68; tongue still parched, and rather brown.

Omit. decoctum senegæ. To sit up for an hour daily.

6th. No stool for two days.

℞. Rhei radicis contritæ gr. xv; hydrargyri submuriatis gr. v. Misce. Sit dosis statim sumenda.

7th. Has had copious evacuations. Bears the sitting up well. Pulse 72; tongue continues obstinately dry; urine quite natural. Other symptoms as before.

He is taking no medicine. To have milk diet.

8th. Pulse 82; bowels regular; tongue still dry; skin natural; appetite good. He wants flesh meat for dinner, but this I have not allowed.

10th. Tongue continues dry.

12th. Tongue continues dry, but somewhat moister in the evening. Has no other symptom of disease, and gets strength daily.

13th. Tongue is now moist over the whole surface

℞. Acidi sulphurici diluti ♏. xxx; zinci sulphatis gr. iv; tincturæ cardamomi compositæ f ʒiij; tincturæ calumbæ f ʒv; infusi calumbæ f ℥vij. Misce. Sumatur f ℥j, ter quotidie.

21st. To omit the milk diet, and have the common diet of the house.

26th. Discharged perfectly well, remarkably hearty, and full of gratitude.

REMARKS.

The principal remark which I have to make, with reference to the foregoing case, is on the great improvement which, notwithstanding the sneers of ignorance, has evidently taken place within a few years even, in the treatment of disease. In the time of Cullen, the above case would have been treated with bark; and blood-letting would have been thought murder. From the vast benefit, however, produced by blood-letting in this instance, we need no ghost to tell us what would have been the effect of an opposite treatment. It would, most probably, have made a ghost of the patient. That I am warranted in saying it would have been treated with bark in the time of Cullen, will be fully shown by the following extract from his writings. Speaking of the cinchona bark, he says,

" Some writers, indeed, mention its salutary effects in various cases of pneumonic inflammation, and such, perhaps may occur; but I never found the bark safe in any such inflammatory affection, except when this was not the primary disease; and, indeed, only when it was accidentally combined with an intermittent, putrid, or nervous fever. That there may be such combinations is well known; and though there may be some degree of phlogistic diathesis present, it may not be in such a degree as to give the principal indications in the cure of the disease: so, in such cases, the bark may be employed as suited to that principal indication."—*Materia Medica*, vol. ii. p. 100.

If I am asked why I bled the man at a time when I was in an unavoidable error as to the state of his pulse, I answer, that I considered the flushed countenance, the suffused conjunctiva, the laborious respiration, the pain in the head and thorax, as surer indications than the pulse; that I was encouraged in my opinion by a judicious practitioner, who happened to be present, and who felt the wrist as well as myself; that I took care to watch the effect of the loss of blood, so as to be able to stop it in a moment, if it seemed to be doing harm;—but, above all, that my plan of treatment was crowned with success—which, in medicine as well as in war, answers all objections.

The long continued and obstinate dryness of the tongue was a remarkable feature in this case, and one for which I do not pretend to account.

The efficacy of the mercurial pill, in restoring the healthy state of the abdominal evacuations, was, in this case, very striking; and I may add, that in almost every case of fever, the use of mercury will be found beneficial: I seldom omit it.

